# Derepression of *LOXL4* inhibits liver cancer growth by reactivating compromised p53

**DOI:** 10.1038/s41418-019-0293-x

**Published:** 2019-02-06

**Authors:** Jialiang Shao, Jiongjiong Lu, Wencheng Zhu, Hua Yu, Xiaoqian Jing, Yi-Lin Wang, Xiang Wang, Xiong-Jun Wang

**Affiliations:** 1 Precise Genome Engineering Center, School of Life Sciences, Guangzhou University, Guangzhou, 510006 China; 2 Department of Urology, Shanghai General Hospital, Shanghai Jiaotong University, Shanghai, 200080 China; 3 Department of Special Treatment and Liver Transplantation, Eastern Hepatobiliary Surgery Hospital, Shanghai, 200438 China; 4 Shanghai Institute of Biochemistry and Cell Biology, Chinese Academy of Sciences, 320 Yue Yang Road, Shanghai, 200031 China; 5 Department of Surgery, Ruijin Hospital, Shanghai Jiao Tong University School of Medicine, Shanghai, 200025 China; 6 Department of Hepatic Surgery, Fudan University Shanghai Cancer Center, Shanghai, 200032 China; 7 Department of Oncology, Shanghai Medical College, Fudan University, Shanghai, 200032 China

**Keywords:** Tumour-suppressor proteins, Tumour-suppressor proteins

## Abstract

*TP53* is the most frequently mutated gene in human cancer, whereas tumors with wild-type *TP53* develop alternative strategies to survive. Identifying new regulators of p53 reactivation would greatly contribute to the development of cancer therapies. After screening the entire genome in liver cancer cells, we identified lysyl oxidase-like 4 (LOXL4) as a novel regulator for p53 activation. We found that 5-azacytidine (5-aza-CR) induces *LOXL4* upregulation, with LOXL4 subsequently binding the basic domain of p53 via its low-isoelectric point region. The interaction between LOXL4 and p53 induces the reactivation of compromised p53, resulting in cell death. Furthermore, the nude mouse xenograft model showed that the 5-aza-CR-dependent LOXL4-p53 axis reduces tumor growth. A positive correlation between LOXL4 expression and overall survival in liver cancer patients with wild-type p53 tumors was observed. In conclusion, we found that 5-aza-CR-induced LOXL4 upregulation reactivates wild-type p53 and triggers cell death, which blocks liver cancer development.

## Introduction

Cancer cells are generally resistant to programed cell death [1]. During carcinogenesis, they develop an adequate program to escape cell cycle control. p53 is a master regulator of cell cycle and is highly mutated in different cancer types [2]. However, in many tumors with wild-type (WT) p53, its functions are repressed at the transcriptional and/or post-transcriptional levels [3, 4]. Thus, reactivation of compromised p53 could benefit many cancer patients.

Liver cancer is the fifth most common cancer and the second leading cause of cancer death worldwide [5, 6]. There are over 780,000 new liver cancer cases and approximately 740,000 deaths worldwide every year [6]. Clinically, a common medical treatment for liver cancer is surgical resection, which is accompanied by a high rate of recurrence [7]. Conventional chemotherapeutic and radiotherapeutic treatments are generally ineffective for liver cancer. For advanced hepatocellular carcinoma, the only approved drug, sorafenib, prolongs overall survival by approximately 3 months [8]. Thus, discovery of novel strategies and drug targets for liver cancer treatment is urgent. In liver cancer, with up to 30–50% of tumors exhibit *TP53* mutations [9-11], which means at least half of liver cancer patients possess tumors with WT, but compromised p53. Therefore, reactivating compromised p53 would be a potential target for liver cancer therapy.

Lysyl oxidase-like 4 (LOXL4) is one of five paralogues in the lysyl oxidase (LOX) family, which includes LOX and LOXL1–4 [12]. The major function of the LOX family is covalent cross-linking of collagens and/or elastin in the extracellular matrix (ECM). Aberrant expression and activity of these proteins have been reported in several cancer types [12-14]. However, the role of LOXL4 in tumor biology remains enigmatic. A few studies have suggested that it promotes tumor proliferation and/or metastasis in head and neck squamous cell carcinoma and gastric cancer [15, 16]. However, in bladder and breast cancer, LOXL4 might function as a tumor suppressor because its loss promotes cancer cell proliferation and metastasis [16, 17]. We speculate that LOXL4 executes its progressive or repressive roles in different tumors depending on tumor cell context and tumor stages. Currently, how LOXL4 functions in liver cancer is not understood.

Here, we found that LOXL4 is a novel regulator that contributes to p53 activation in liver cancer. 5-azacytidine treatment upregulated *LOXL4* expression, leading to LOXL4 binding with p53, which increased p53 phosphorylation at serine 15 and resulted in p53 activation. Disruption of the LOXL4-p53 axis promoted tumor cell proliferation, whereas enhanced LOXL4-p53 interaction strongly reduced tumor cell growth both in vitro and in vivo. Together, our results illustrate that 5-azacytidine-dependent *LOXL4* derepression functionally contributes to the activation of compromised p53, which offers a promising therapeutic strategy for liver cancer.

## Results

### A genome-wide CRISPR screen identified LOXL4 as a novel regulator of 5-aza-CR-dependent cell death

**Fig. 1:**
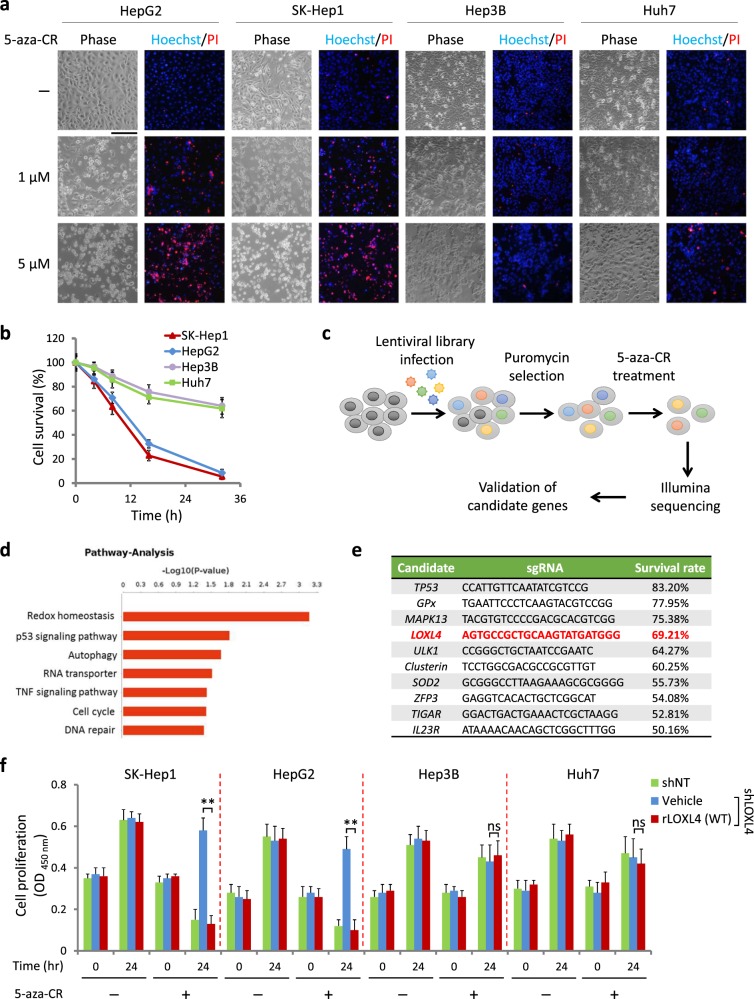
A genome-wide CRISPR screen identified LOXL4 as a novel regulator of 5-aza-CR-dependent cell death. **a** Live and dead cell imaging after Hoechst 33324 and propidium iodide (PI) double staining. Cells were treated with or without 5-aza-CR (1 or 5 μM) for 24 h and then double stained for 0.5 h. Scale bar: 100 μm. Experiments were independently performed three times. **b** Survival rates of HepG2, Huh7, Hep3B, and SK-Hep1 cells in response to 5-aza-CR treatment. Cells were treated with 5-aza-CR (5 μM) for different lengths of time: 0, 4, 8, 16, and 32 h, followed by trypan blue staining. The survival rates of living cells were calculated using Life Tech (Invitrogen) Countness^R^. Data were from three independent experiments performed in triplicate; error bars represent SEM. **c** Workflow of lenti-CRISPR/cas9 screening for genes required for 5-aza-CR-induced cell death. The five key steps included in this workflow are as follows: (1) lentiviral library infection of SK-Hep1 cells for 2 days; (2) selection of efficiently infected cells using puromycin (5 μg/mL); (3) 5-aza-CR (5 μM) treatment for 2 days; (4) extraction and sequencing of the genomes of surviving cells using an Illumina sequencer with a HiSeq instrument followed by analysis with BaseSpace Sequence Hub; (5) validation of candidate genes by evaluating the survival rate over 50% after 5-aza-CR (5 μM) treatment for 2 days. **d** Enriched genes. The sequencing results were analyzed and summarized in Gene Ontology terms using David GO. **e** The top 10 genes contributing to 5-aza-CR-induced cell death. The genes were selected based on a survival rate of over 50% after 5-aza-CR (5 μM) treatment for 2 days after knockout of these genes. Experiments were independently performed three times. **f** Cell proliferation during 5-aza-CR treatment. Cells were treated with 5 μM 5-aza-CR for 24 h. CCK-8 assays were used to assess cell proliferation with and without treatment. Stable cell lines were created by reintroducing vehicle or LOXL4 into shLOXL4 cells. Data were from three independent experiments performed in triplicate; error bars represent SEM; **p < 0.01

5-azacytidine (5-aza-CR) is a small molecule that induces DNA damage and is primarily used in clinic for treatment of myelodysplastic syndrome [18, 19]. To measure the effect of 5-aza-CR on liver cancer cells, we tested four cell lines (HepG2, SK-Hep1, Hep3B, and Huh7) using Hoechst and propidium iodide (PI) double staining. As shown in Fig. 1a, a low dose (1 μM) of 5-aza-CR-induced substantial cell death in HepG2 and SK-Hep1 cells, while an even higher dose (5 μM) caused no obvious damage to either Hep3B or Huh7 cells. Next, we measured cell survival across different time points. As shown in Fig. 1b, the survival rates of HepG2 and SK-Hep1 cells were close to zero, while Huh7 and Hep3B cells exhibited greater than 60% survival after 32 h of treatment. Furthermore, 5-aza-CR treatment induced both apoptosis and necrosis in HepG2 and SK-Hep1 cells, but not in Hep3B and Huh7 cells (Fig. S1).

To determine which genes are involved in 5-aza-CR-dependent cell death, we applied lenti-CRISPR/Cas9 plasmid-containing lentivirus [20], which knocks out genes from the genome for screening purposes. To reduce the proportion of false positives, SK-Hep1 cells were first treated with 5 μM 5-aza-CR for two days and then collected for DNA extraction and genome sequencing (Fig. 1c). With an evaluation criterion of a survival rate over 20% under 5-aza-CR treatment, 28 candidates were identified by sequencing, and their enriched pathways are summarized in Fig. 1d. By elevating the survival rate to 50%, we determined that ten of the 28 candidates were important for cell death (Fig. 1e). Among them, *TP53* and *GPx* have been reported as a key regulator and an enzyme that mediate cell death and control intracellular ROS levels, respectively [21-24]. *MAPK13*, also known as *p38delta*, is a member of the p38 kinase family, which is involved in cell death [25]. *LOXL4* has not been reported to participate in cell death regulation yet (Fig. 1e).

We next sought to determine the role of *LOXL4* in mediating the effects of 5-aza-CR treatment on cell death. We found that loss of *LOXL4* reduced the induction of cell death under 5-aza-CR treatment in SK-Hep1 and HepG2 cells, but not in Hep3B and Huh 7 cells (Fig. 1f). The knockdown efficacy of *LOXL4* was determined by western blotting (Fig. S2). Thus, *LOXL4* seems to be an important factor involving in 5-aza-CR-induced cell death in SK-Hep1 and HepG2 cells.

### Forced expression of LOXL4 induces cell death in SK-Hep1 and HepG2 cells but not in Hep3B and Huh7 cells

**Fig. 2:**
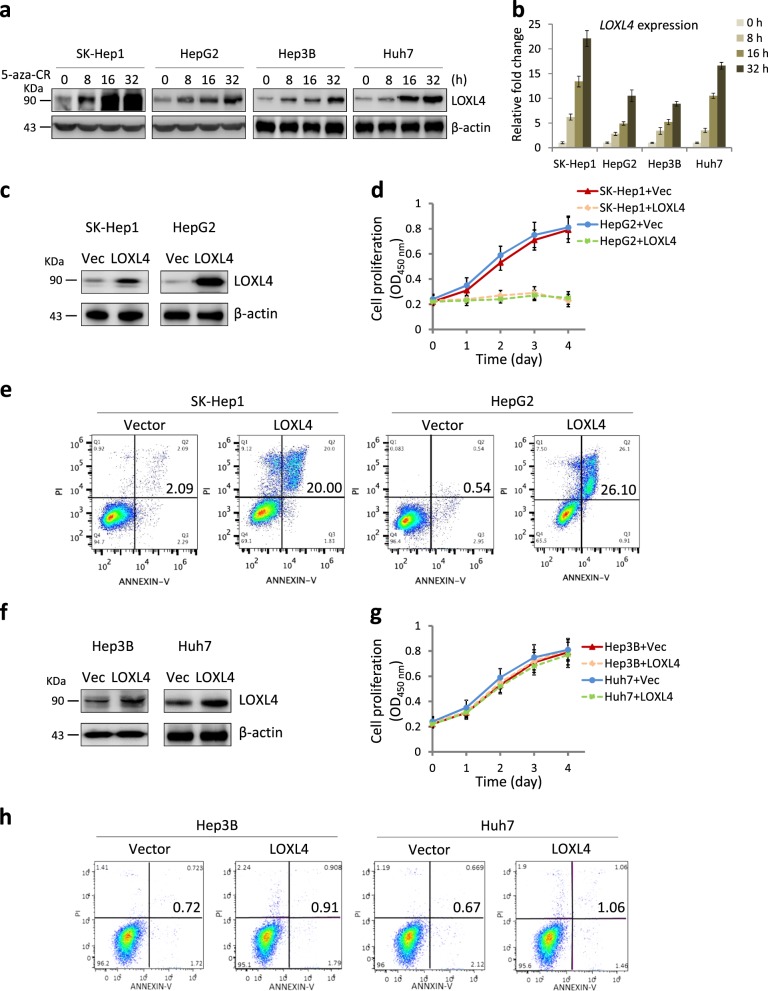
Forced LOXL4 expression efficiently induces apoptosis in HepG2 and SK-Hep1 cells but not in Hep3B and Huh7 cells. **a, b** 5-aza-CR treatment upregulates LOXL4 expression. Cells were treated with 5-aza-CR (5 μM) for different lengths of time: 0, 8, 16, and 32 h and then analyzed via western blotting (**a**) or qRT-PCR (**b**). Data were from three independent experiments performed in triplicate; error bars represent SEM. **c**, **f** Forced LOXL4 expression in SK-Hep1, HepG2, Hep3B, and Huh7 cells. Lentivirus were used to infect cells followed by puromycin (50 μg/mL) selection. The efficiency of LOXL4 expression was confirmed by western blotting using the indicated antibodies. **d, g** Proliferation of SK-Hep1, HepG2, Hep3B, and Huh7 cells from (**c**) and (**f**). CCK-8 assays were used to measure cell proliferation at the indicated time points. Data were from three independent experiments performed in triplicate; error bars represent SEM. **e**, **h** FACS analysis of SK-Hep1, HepG2, Hep3B, and Huh7 cells from (**c**) and (**f**) after PI and anti-Annexin V double staining. Cells were treated with 5-aza-CR (5 μM) for 16 h before collection and then the FACS analysis was performed according to the operation manual of the PI and anti-Annexin V double staining kit. Experiments were independently performed three times

As a well-known DNA methyltransferase (DNMT) inhibitor, 5-aza-CR may be involved in the regulation of *LOXL4* expression. First, we detected the LOXL4 protein levels during 5-aza-CR treatment. As shown in Fig. 2a, LOXL4 gradually accumulated in the four cell lines in response to 5-aza-CR. Quantitative real time polymerase chain reaction (qRT-PCR) further revealed that the observed LOXL4 accumulation depended on its upregulated transcription (Fig. 2b). A previous study indicates that another DNMT inhibitor 5-aza-2′-deoxycytidine also can epigenetically upregulates *LOXL4* transcription in bladder cancer cells [26].

To further verify the inhibitory effect of LOXL4, we constructed cell lines that stably expressed LOXL4. The LOXL4 expression in the forced expression group was 5–10 times higher than that in the empty vector group (Fig. 2c, f). Forced expression of LOXL4 dramatically decreased HepG2 and SK-Hep1 cell growth to the same extent (Fig. 2d). However, enhanced LOXL4 expression did not inhibit either Hep3B or Huh7 cells (Fig. 2g). This result prompted the question of how LOXL4 influences cell growth. Combined with the findings shown in Fig. 1a, we speculated that LOXL4 induced cell growth arrest by triggering apoptosis signaling. As expected, forced expression of LOXL4 elevated apoptosis levels from 2.09 to 20.0% (for SK-Hep1 cells) and from 0.5 to 26.1% (for HepG2 cells) (Fig. 2e), but not in Hep3B and Huh7 cells (Fig. 2h). Therefore, accumulated LOXL4 can trigger cell death in SK-Hep1 and HepG2 cells.

### LOXL4 interacts with WT p53, which is required for 5-aza-CR-induced cell death

**Fig. 3:**
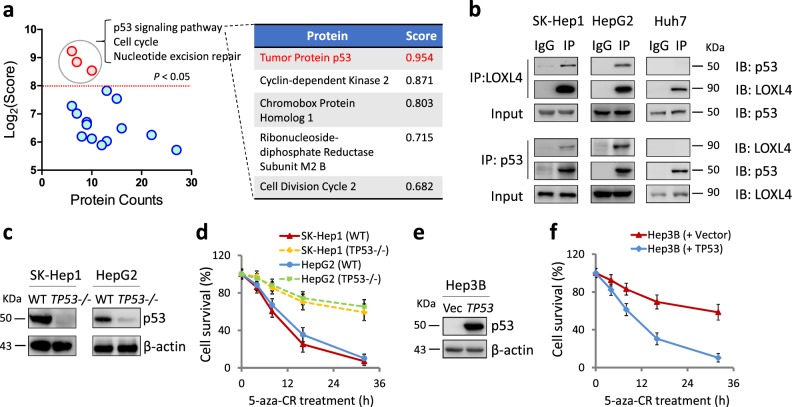
LOXL4 interacts with p53, which is required for 5-aza-CR-induced cell death. **a** Gene Ontology terms among LOXL4 interaction partners identified in SK-Hep1 cells after 5-aza-CR (5 μM) treatment for 8 h. The samples in this figure were prepared with a specific anti-LOXL4 antibody. The score was determined using the web software, David GO. *p* values, Benjamini–Hochberg test. Proteins with *p* < 0.05 and log_2_ (Score) > 8.0 indicated proteins with significantly increased interaction with LOXL4 after 5-aza-CR treatment. **b** Endogenous interaction between LOXL4 and p53 under 5-aza-CR (5 μM) treatment for 8 h. LOXL4 or p53 immunoprecipitates were analyzed with antibodies recognizing p53 or LOXL4. **c** Knockout of the *TP53* gene in SK-Hep1 and HepG2 cells. CRSPR-Cas9-mediated knockout of *TP53* by sgRNA. The sgRNA sequence in the target responding genomics was identified by sequencing. **d** Survival rates of SK-Hep1, HepG2 wild-type (WT) or *TP53* gene knockout (*TP53*^*−/−*^) cells in response to 5-aza-CR (5 μM) treatment at the indicated time points. Cell survival was measured by trypan blue staining. Data were from three independent experiments performed in triplicate; error bars represent SEM. **e** Forced expression of *TP53* in Hep3B cells. The p53 protein level was detected by western blotting. **f** Survival rate of Hep3B cells (Vector or *TP53* gene overexpression) in response to 5-aza-CR (5 μM) treatment at the indicated time points. Cell survival was measured by trypan blue staining. Data were from three independent experiments performed in triplicate; error bars represent SEM

5-aza-CR-induced LOXL4 accumulation was observed in four cell lines, but their differential responses to 5-aza-CR indicates that the function of LOXL4 in cell death involves other proteins. To identify associated partners or substrates of LOXL4, LOXL4-associated proteins were immunoprecipitated from SK-Hep1 cells treated with or without 5-aza-CR and were subjected to tandem mass spectrometry analysis. Gene ontology analyses of LOXL4 interactors identified from SK-Hep1 cells revealed that the p53 signaling pathway, cell cycle and nucleotide excision repair were the only significantly enriched functions (*p* < 0.05, Fig. 3a). Indeed, p53 ranked at the top, scoring 0.954 among the top five proteins involved in the p53 signaling pathway that bound to LOXL4 (Fig. 3a). The predicted p53 protein is marked in the sodium dodecyl sulfate polyacrylamide gel electrophoresis (SDS-PAGE) gel shown in Fig. S3a. Peptide number and coverage percentage are listed in Fig. S3b. These results suggest that p53 is a likely candidate for LOXL4 interaction.

Next, we transfected HEK293 cells with FLAG-LOXL4 and HA-p53 and found an interaction between LOXL4 and p53 (Fig. S3c). Co-immunoprecipitation (Co-IP) experiments with endogenous LOXL4 and p53 confirmed this interaction in HepG2 and SK-Hep1 cells, both of which contain WT p53, but not in Huh7 cells, which contain mutated p53 (Fig. 3b). We speculated that the differential sensitivities of four cell lines to 5-aza-CR may be related to the p53 status in each cell line. Indeed, p53 knockout in SK-Hep1 and HepG2 cells made them resistant to 5-aza-CR (Fig. 3c, d), whereas overexpression (OE) of WT p53 in Hep3B cells (*TP53* null) dramatically increased cell death under 5-aza-CR treatment (Fig. 3e, f). Furthermore, we tested how *TP53* knockout (KO) or OE would affect cell proliferation without 5-aza-CR treatment. As shown in Fig. S4, *TP53* KO can slightly enhance cell growth, while *TP53* OE partly inhibits cell growth. Of note, *LOXL4* is not a p53 transcriptional target gene as *TP53* overexpressing does not alter either transcription level or protein level of LOXL4 (Fig. S5). Thus, the reversed cell death sensitivity in these cell lines depends on the function of p53. Classically, LOXL4 is a secreted protein for ECM remodeling. We applied immunofluorescence staining to detect the cellular localization of LOXL4 in SK-Hep1 and HepG2 cells. Before 5-aza-CR treatment, LOXL4 is weakly formed spot-like fluorescence (Fig. S6). After the treatment, LOXL4 is evidently increased and distributed in the cytoplasm similar to a previous report [27]. Our findings suggest that overexpressed LOXL4 interacts with WT p53 which is required for promoted cell death.

### Anion and cation bonding dictates the interaction between LOXL4 and p53

**Fig. 4:**
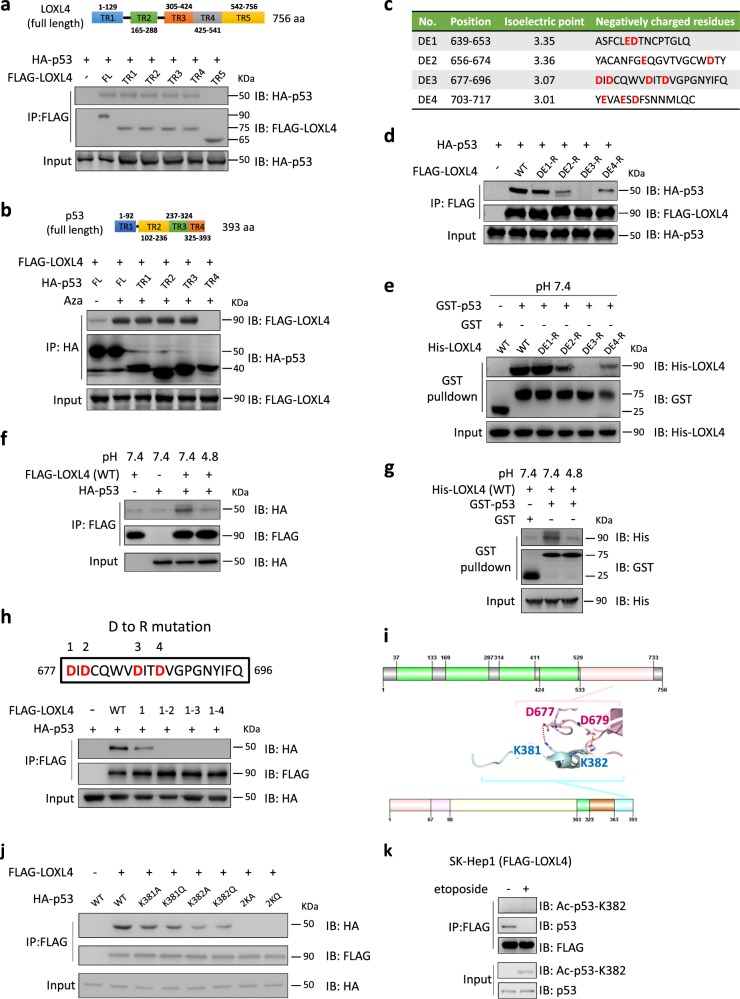
Ionic bonding between anions and cations determines the interaction between LOXL4 and p53. **a** SK-Hep1 cells were co-transfected with FLAG-LOXL4 (full-length and five truncated forms) and HA-p53. After 48 h, cell lysates were enriched with FLAG antibody and analyzed using the indicated antibodies. TR1–TR5 indicate the truncated regions from the full-length protein. **b** SK-Hep1 cells were co-transfected with HA-p53 (full-length and four truncated forms) and FLAG-LOXL4. After 48 h, cell lysates were enriched with HA antibody and analyzed using the indicated antibodies. TR1–TR4 indicate the truncated regions from the full-length protein. **c** Screening for the region containing an isoelectric point less than 3.5 located in LOXL4- C terminal of 542–756 amino acids and mutation of all Asp (D) or Glu (E) residues to Arg (R). Region DE1, from 639 to 653 aa; Region DE2, from 656 to 674 aa; Region DE3, from 677 to 696 aa; Region DE4, from 703 to 717 aa. **d** SK-Hep1 cells were transfected with FLAG-LOXL4 (including DE1-R to DE4-R mutants) plasmids. After 48 h, cell lysates were enriched with FLAG antibody and analyzed using the indicated antibodies. DE1 mutation to Arg (R), DE1-R; DE2 mutation to Arg (R), DE2-R; DE3 mutation to Arg (R), DE3-R; DE1 mutation to Arg (R), DE4-R. **e** GST pull-down assay to detect a direct interaction between LOXL4 and p53 at pH 7.4. **f** SK-Hep1 cells were co-transfected with FLAG-LOXL4 and HA-p53. After 48 h, cell lysates with different pH values were enriched with FLAG antibody and analyzed using the indicated antibodies. **g** GST pull-down assay to detect a direct interaction between LOXL4 and p53 at pH 7.4 or pH 4.8. **h** Screening of functional D residues in LOXL4 that are required for binding p53. D677 mutation to R677, 1 mutant; D677/679 mutation to R677/679, 1–2 mutant; D677/679/684 mutation to R677/679/684, 1–3 mutant; D677/679/684/687 mutation to R677/679/684/687, 1–4 mutant. **i** Docking simulation between LOXL4-DE3 and p53-C terminal region. The ionic bonding between D677/679 in LOXL4 and K381/382 in p53 is shown. The structure of p53 from 367aa to 388aa was obtained from a pdb file (accession number: 1DT7). The structure of Loxl4 was predicted using RaptorX. **j** SK-Hep1 cells were co-transfected with FLAG-LOXL4 and HA-p53 (WT or mutant). After 48 h, cell lysates were enriched with FLAG antibody and analyzed using the indicated antibodies. Experiments were independently performed three times. **k** SK-Hep1 cells stably expressing FLAG-LOXL4 (treated with 2 μM of etoposide for 6 h or untreated) were enriched with FLAG antibody and analyzed using the indicated antibodies. Experiments were independently performed three times

To identify how LOXL4 interacts with p53, we created LOXL4 and p53 truncations, as shown in Fig. 4a, b, respectively. Analysis of LOXL4 truncations showed that deletion of the C terminal 542–756 amino acids (aa) abolished the interaction with p53 (Fig. 4a). When the C-terminal region of p53 that contains enriched clusters of basic aa (325–393) was absent, p53 lost its interaction with LOXL4. In the p53 basic domain (containing 33 amino acids), 11 lysine (Lys), or arginine (Arg) residues present strong positive charges. We screened the C-terminal region of LOXL4 (TR5), which presents strong negative charges, containing an isoelectric point less than 3.5 (Fig. 4c). Mutating four aspartic acid (Asp) residues to Arg residues in the 677–696 aa region (DE3) almost completely disrupted the interaction between FLAG-LOXL4 and HA-p53. In the 656–674 aa (DE2) and 703–717 aa (DE4) regions, mutation of Asp or glutamic acid (Glu) residues to arginine (Arg) led to a moderately decreased interaction (Fig. 4d), indicating that the 656–717 aa region is essential for targeting the basic domain of p53. GST pull-down assays with phosphate-buffered saline (PBS, pH = 7.4) showed results similar to those of the in vivo co-immunoprecipitation between LOXL4 and p53 (Fig. 4e), while disodium hydrogen phosphate-citrate buffer (pH = 4.8) used in the Co-IP (Fig. 4f) or GST pull-down (Fig. 4g) assays nearly abolished the interaction between LOXL4 and p53, indicating that their direct interaction depends on the force of cations and anions between the LOXL4 677–700 aa region and the p53 basic domain. Further, four mutants of FLAG-LOXL4 (Fig. 4h, upper panel) were constructed to identify which Asp residues are required for binding p53, and the results showed that the D677/679R mutation completely blocked the interaction with p53 (Fig. 4h, lower panel). Next, we performed a molecular docking simulation using the program PYMOL and found that K381/382 in p53 are two positively charged residues that bind to D677/679 in LOXL4 (Fig. 4i). To confirm this, we co-expressed FLAG-LOXL4 and HA-p53 (WT or mutant) in SK-Hep1 cells. The Co-IP experiments demonstrated that mutation either of two sites in p53 would reduce its interaction with LOXL4 and mutation of both sites completely abolished the interaction between p53 and LOXL4 (Fig. 4j). These data suggest that binding of LOXL4 to p53 results in neutralization of the positive charge in p53 provided by the K381/382 residues. Lysines in the C-terminal regulatory domain of p53, such as K370, K372, K373, K381, K382, and K386, are regularly acetylated by p300/CBP, and acetylation of these lysines induces p53 activation [28]. We speculated that LOXL4 may play a role similar to that of p300/CBP in activating compromised p53. How would the LOXL4 bound p53 respond to K381/382 acetylation? Etoposide induced DNA damage is known to trigger p53 acetylation at K381/382 [29]. Our data showed that binding by LOXL4 reduced the K382 acetylation of p53 during DNA damage in SK-Hep1 cells stably expressing LOXL4 (Fig. 4k). Therefore, binding with LOXL4 and acetylation at K381/382 are likely two parallel ways to promote p53 activation which also antagonizes with each other.

### The LOXL4-p53 interaction is required for p53 activation and cell death in response to 5-aza-CR treatment

**Fig. 5:**
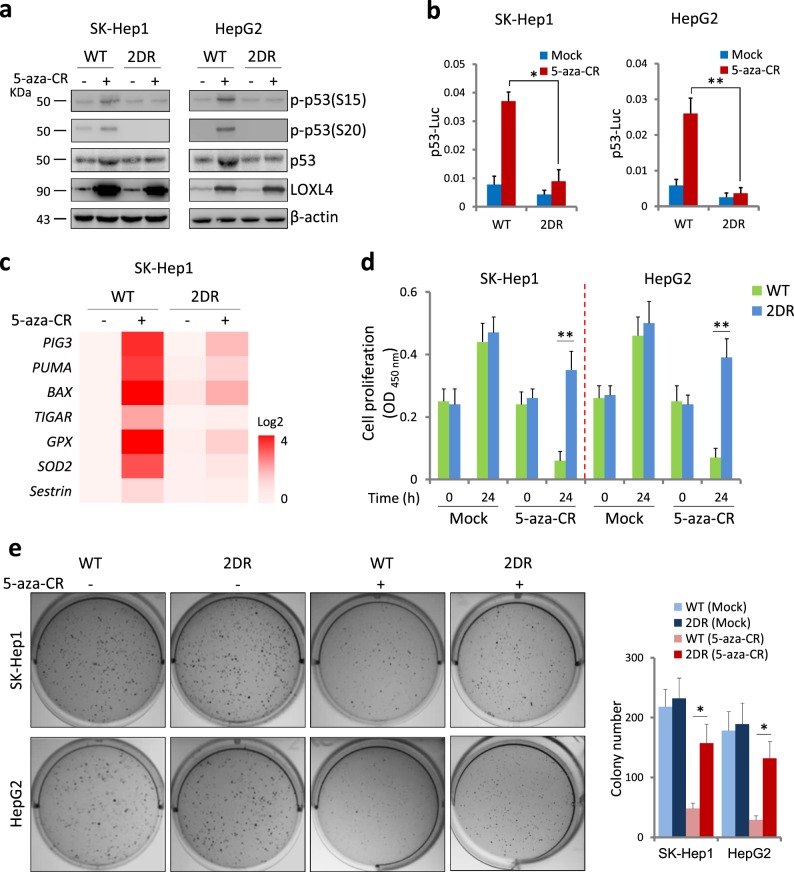
LOXL4 is required for p53 activation, which depends on LOXL4-p53 interaction. **a** Western blotting analysis of cells with wild-type or 2DR (D677/679R) mutated *LOXL4* in response to 5-aza-CR (5 μM) treatment for 24 h. The strategies for creating D677/679R (2DR) mutant LOXL4 in SK-Hep1 and HepG2 cells using CRISPR/Cas9 are described in Fig. S7. The indicated antibodies were used to examine LOXL4 protein levels and p53 activation. Experiments were independently performed three times. **b** 2DR mutation abolished p53 activation in response to 5-aza-CR (5 μM) treatment for 24 h. The cell lines used to measure p53-luciferase reporter activity are originated from Fig. 5a. Data were from three independent experiments performed in triplicate; error bars represent SEM. **c** Gene expression induced by 5-aza-CR (5 μM) treatment for 16 h. Several downstream p53 target genes were detected via quantitative real time PCR. Experiments were independently performed three times. **d** Cell proliferation of SK-Hep1 and HepG2 cells with or without 5-aza-CR (5 μM) treatment. A CCK-8 assay was used to measure cell proliferation. Data were from three independent experiments performed in triplicate; error bars represent SEM. **e** Colony formation of SK-Hep1 and HepG2 cells with or without 5-aza-CR (2 μM) treatment for two weeks. The total number of colonies in a six-well plate was calculated using ImageJ software (version 1.8.0). Data were from three independent experiments performed in triplicate; error bars represent SEM

DNA damage-induced p53 activation is marked by phosphorylation of p53 serine 15 (S15) and serine 20 (S20) [30-32]. To examine the effect of the LOXL4-p53 axis formation on p53 activation, we created a D677/679R (2DR) mutant LOXL4 in SK-Hep1 and HepG2 cells using CRISPR/Cas9 (Fig. S7). As shown in Fig. 5a, WT LOXL4 cells responded to 5-aza-CR, leading to increased p53 phosphorylation at S15 and S20. Disruption of this interaction by 2DR mutant did not elevate p53 phosphorylation. Luciferase assays using a p53-responsive luciferase reporter vector showed that p53 transcriptional activity is dependent on LOXL4-p53 axis formation (Fig. 5b).

Dramatically elevated activation of p53 can induce apoptosis or necrosis. To examine the functional consequences of the LOXL4-p53 axis, we measured the expression of p53 downstream genes. As shown in Fig. 5c, the expression of *PIG3* and *BAX*, which induce apoptosis, increased over tenfolds in WT cells but not in 2DR cells. *GPx1* and *SOD2* were also dramatically upregulated with p53 activation, while the expression levels of *TIGAR* and *SESTRIN1* remained unchanged (Fig. 5c). Moreover, WT and 2DR cells showed similar growth rates under normal growth conditions; 2DR cells were however resistant to 5-aza-CR, indicating that LOXL4 binding to p53 is required for cell death (Fig. 5d). Colony formation experiments further confirmed that 5-aza-CR-dependent cell death requires the LOXL4-p53 interaction in both SK-Hep1 and HepG2 cells (Fig. 5e). These results suggest that LOXL4 could elevate p53 activation by binding with p53, while 2DR mutant of LOXL4 which lost its interaction with p53 is unable to promote p53-dependent cell death.

### 5-aza-CR inhibits tumor growth through the LOXL4-p53 interaction in vivo

**Fig. 6:**
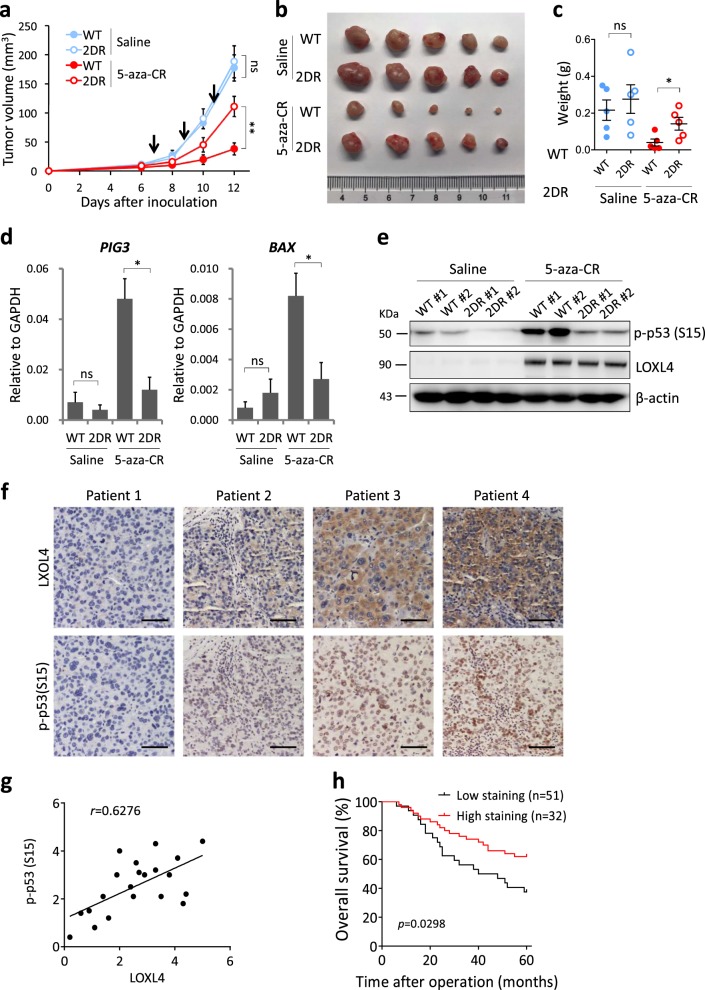
The LOXL4-p53 interaction for xenograft growth and patient survival. **a** Calculation of tumor volume from inoculation to sacrifice of the mice. *n* = 5; error bars represent ± SEM. Five Balb/c nude mice were injected in the left groin with 2 × 10^6^ SK-Hep1 cells. 5-aza-CR at a dose of 5 nmol/g weight (a quarter of the normal clinical dose) was injected into mice twice a day for three sequential days. Saline was used as a negative control for 5-aza-CR. Tumor size was measured every three days. The arrows indicate drug administration time points. **b** A representative image of tumors. **c** Tumors were weighed after the mice were sacrificed. *n* = 5; error bars represent SEM. **d** *PIG3* and *BAX* expression in xenograft tumors. All five xenograft tumors were collected for RNA extraction and to measure *PIG3* and *BAX* expression in the four groups. *n* = 5; error bars represent SEM. **e** Western blotting analysis of two representative tumors from Fig. 6b using antibodies against LOXL4 and p-p53 (S15). **f** Representative images of immunohistochemical staining of tumor tissues from four patients using antibodies against LOXL4 and p-p53 (S15); the two antibodies were diluted at 1:100 and 1:100, respectively. **g** Statistical analysis of LOXL4 and p-p53 (S15) staining in 21 matched human liver cancer tissues. Statistical significance was assessed using Fisher’s exact test. LOXL4 was positively correlated with the signal intensity of p-p53 (S15), with *r* = 0.6276 and *p* < 0.001. **h** Overall survival rates of 83 liver cancer patients with different LOXL4 staining intensities were compared. LOXL4 negatively regulates liver cancer development and patient survival rates (*p* = 0.0298)

To evaluate the effects of the LOXL4-p53 axis on tumor growth in vivo, SK-Hep1 cells with WT LOXL4 or mutant LOXL4 (2DR) were implanted into nude mice. Twenty BALB/c nude mice were divided into four groups: WT + saline; 2DR + saline; WT + 5-aza-CR; 2DR + 5-aza-CR. As illustrated in Fig. 6a, 6 days after tumor inoculation, we systemically administered saline or 5-aza-CR every two days for a total of three treatments. The results showed that WT and 2DR tumors treated with saline displayed similar growth rates in vivo (Fig. 6a, b). However, upon 5-aza-CR treatment, the growth of WT tumors was significantly reduced, whereas 2DR tumors showed slight growth inhibition. After 5-aza-CR treatment, the average weight of WT tumors was 0.04 ± 0.02 g, while that of 2DR tumors was 0.14 ± 0.03 g (Fig. 6c). We also examined downstream targets of p53 activation, *PIG3* and *BAX* expression, from the same samples in Fig. 6b. These two genes showed strong increases upon 5-aza-CR treatment in WT, but not 2DR tumors (Fig. 6d). To confirm p53 activation, two tumor samples from each group in Fig. 6b were examined by western blotting. Upon 5-aza-CR stimulation, p53 activation was observed at higher levels in WT than in 2DR tumors (Fig. 6e). These results suggest that the 5-aza-CR triggered LOXL4-p53 axis is required for repressing xenograft growth.

### The LOXL4 protein level is correlated with p53 activation, tumor development, and liver cancer patient survival

To define the clinical relevance of LOXL4 in liver cancer patient survival, we analyzed human liver cancer tissues by immunohistochemistry (IHC). First, 21 liver cancer patient tissues (WT *TP53*) were randomly selected for staining with antibodies against LOXL4 and p-p53 (S15). The LOXL4 protein levels in human liver cancer tissues with WT *TP53* were tightly correlated with p53 activation, along with increased p53 S15 phosphorylation (Fig. 6f). Statistically, the LOXL4 level displayed a significant positive correlation with p-p53 (S15) (*p* < 0.001, *r* = 0.6276) (Fig. 6g). Statistical analysis of clinical samples containing WT p53 showed that LOXL4 decreased by degrees in conjunction with falling survival rates in liver cancer patients (*p* = 0.0298) over 5 years (Fig. 6h).

### LOXL4-mediated p53 activation may be relevant in other cancer types

**Fig. 7:**
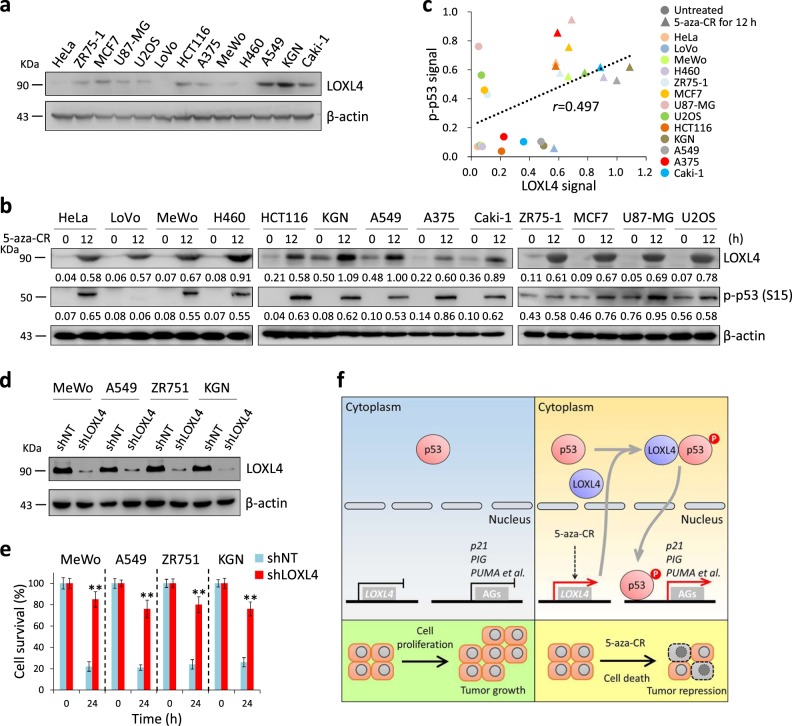
LOXL4/p53 axis in other cancer types. **a** LOXL4 was detected in indicated cell lines by western blotting. Experiments were independently performed three times. **b** Western blotting analysis of cell lysate from indicated cell lines before and after 5 μM 5-aza-CR treatment. Experiments were independently performed three times. **c** Correlation between LOXL4 and p-p53 (S15) intensities from indicated cell lines; **d** LOXL4 knockdown in indicated cell lines. The cell lysate was analyzed by western blotting. **e** Cell survival under 5 μM 5-aza-CR treatment for 24 h. Cells were seeded in 96-well plate at a density of 3000 cells/well. Twenty-four hour later, the cell survival was measured by CCK-8 and was set as 0 h. Then cells were treated with PBS (control) or 5-aza-CR for another 24 h. The cell survival was measured by CCK-8, and the survival ratio at 24 h is a relative value to the untreated group (PBS). Data were from three independent experiments performed in triplicate; error bars represent SEM. **f** The proposed mechanism of LOXL4-p53 axis-regulated cell death

Herein, our results demonstrate that silenced LOXL4 can be restored by 5-aza-CR, which in turn enhances p53 activation. To test whether this mechanism is relevant in other cancer types with WT p53, we collected 13 cell lines from 10 types of solid tumors. We detected the LOXL4 protein levels in these 13 cancer cell lines and found that only KGN cells exhibited moderate LOXL4 protein levels, while the other cell lines had low levels of LOXL4 (Fig. 7a). Next, we treated cells with 5 μM 5-aza-CR for 12 h and measured both LOXL4 protein level and p53 phosphorylation. Surprisingly, LOXL4 protein was strongly increased after 5-aza-CR treatment, and p-p53(S15) was accordingly observed in most of the cell lines (Fig. 7b). We calculated the correlation between LOXL4 and p53 S15 phosphorylation and obtained *r* = 0.497, indicating that, to some extent, LOXL4 protein level is positively correlated with p53 activation (Fig. 7c). Next, we selected four cell lines (MeWo, A549, ZR751, and KGN) with higher correlations between LOXL4 and p-p53(S15) to test how LOXL4 knockdown affects cell proliferation under 5-aza-CR treatment (Fig. 7d). As illustrated in Fig. 7e, when LOXL4 decreased, these cells become resistant to 5-aza-CR-induced cell proliferation inhibition. Therefore, our results suggest that the LOXL4/p53 axis might be functional in other tumor types.

Attenuated cell death caused by alterations in p53 function has been documented in human cancers. However, the mechanism by which tumor cells escape from the control of WT p53 remains elusive. As shown in Fig. 7f, we propose a mechanistic model of how tumors respond to cell death signals that specifically integrate the LOXL4 interaction with p53. In tumors with WT p53, *LOXL4* is somehow silenced at the transcriptional level, and 5-aza-CR derepresses *LOXL4*, which subsequently leads to the accumulated LOXL4 reactivates compromised p53, resulting to cell death and tumor regression.

## Discussion

p53 has been investigated for more than 30 years and is regarded as the most important safeguard of genomic stability. Normally, p53 levels in tumors are kept low by the major antagonist, Mdm2, an E3 ubiquitin ligase that is itself a transcriptional target of p53 [33-35]. Acetylation of K381/382 at p53 are proven to prevent Mdm2 binding with p53 [36], resulting in p53 accumulation and consequent activation [37]. In this work, we found that LOXL4 can be elevated by 5-aza-CR and the accumulated LOXL4 in cytoplasm can bind with the cytoplastic p53 [38] through ionic bonds formed by LOXL4 D677/679 residues and p53 K381/382 residues. We propose that LOXL4 may play a similar role as K381/382 acetylation in stabilizing p53. p53 constantly shuttles between the nucleus and the cytoplasm by its nuclear localization signal and nuclear export signals sequences [39]. Thus, the accumulated p53 through LOXL4 binding could enter the nucleus and become active like the inhibition of Mdm2 [37].

WT p53 is frequently silenced or inactivated in cancer cells, such as HepG2 and SK-Hep1 cells. Even though in these tumor cells, p53 still exhibits low levels of transcriptional activity because its KO can further enhance cancer cell growth. Thus, reactivating silenced WT p53 is a potential strategy to conquer liver cancer and other kinds of cancers. Here, we discovered a new activator, LOXL4, whose transcription is likely blocked by DNA methylation in its promoter [26], that can target and reactivate WT p53. This is a feasible scheme for treatment of liver tumors that contain WT *TP53*. Moreover, we found that many cancer cell types containing WT p53 are also sensitive to 5-aza-CR, which could extend the application of 5-aza-CR to multiple solid tumors, such as skin, lung, breast, and ovarian tumors.

Together, our results show that in liver cancer with WT p53, LOXL4 can be defined as a tumor suppressor by regulating p53 activation. Of note, LOXL4 might be considered a double-edged sword during cancer initiation, progression and metastasis due to the balance between the LOXL4-p53 axis and the lysyl oxidase activity of LOXL4 in the matrix. In tumor initiation, silence of LOXL4 can release tumor cells from p53-dependent cell growth inhibition. In contrast, during tumor progression, p53 might lose its function, and upregulation of LOXL4 can promote ECM remodeling to facilitate tumor metastasis. Therefore, our findings reveal one of detailed mechanisms that can explain why WT p53 in some tumors does not exhibit tumor suppressing effects.

## Materials and methods

### Materials

*Cell lines:* HepG2, Hep3B, SK-Hep1, HEK293, HEK293T, HeLa, ZR75-1, MCF7, U87-MG, U2OS, LoVo, HCT116, A375, MeWo, H460, A549, and Caki-1 cell lines were purchased from American Type Culture Collection (ATCC, USA); Huh7 and KGN cell lines were obtained from the Cell Bank of Type Culture Collection of Chinese Academy of Sciences China (Shanghai, China).

*Plasmids*: All of Flag-tagged plasmids are constructed in pCDNA3.0 vector. The lentivirus plasmids for overexpressing *LOXL4* or its mutants are constructed in pCDH vector. Primers used to construct these above plasmids were listed in Supplementary material.

*Antibodies*: Antibodies of anti-HA (sc-7392), anti-LOXL4 (sc-66952), and anti-β-actin C4 (sc-47778) were purchased from Santa Cruz Biotechnology, Inc. (USA); anti-Flag M2 antibody (F1804) and anti-GST antibody (G7781) were purchased from Sigma-Aldrich (USA); anti-p-p53(S15) (#9284), anti-p-p53(S20) (#9287) and anti-acetyl-p53 (Lys382) (#2525) antibodies were purchased from cell signaling technology (USA); anti-p-p53 (S15) (# NB100–92601) was purchased form Novus (USA).

### Cell culture and transfection

HepG2, ZR75-1 and H460 cells were cultured in RPMI-1640 medium; Huh7, Hep3B, SK-Hep1, HEK293, HEK293T, HeLa, MCF7, U87-MG, A375, MeWo, and KGN cells were cultured in DMEM; U2OS, HCT116, and Caki-1 cells were cultured in McCoy’s 5A modified medium; LoVo and A549 cells were cultured in Ham’s F-12K medium; all culture medium were supplemented with 10% fetal calf serum (Gibco). Cell transfection was performed with lipofectamine^TM^2000 (Invitrogen, USA), according to the manufacturer’s instructions.

### Cell proliferation assay

A total of 2 × 10^4^ cells was plated and counted 2 days after seeding in culture medium with 0.5% bovine calf serum. Cell viability under different treatment condition was measured using Cell Counting Kit-8 (CCK-8, Dojindo Laboratories, Japan) according to the manufacturer’s instructions. The cells were plated, in triplicates, at a density of 10,000 cells/well in a volume of 300 μL in 48-well plates. On the following day, 30 μL of the CCK-8 cell-counting solution was added to each well and incubated at 37°C for 3 h. The absorbance of the solution was read spectrophotometrically at 450 nm with a reference at 650 nm using a microtiter plate reader (Becton-Dickinson), which was used as an indicator of cell viability.

### Quantitative real-time PCR

Total RNA was prepared from the cell samples using Trizol (Invitrogen, USA) according to the manufacturer’s protocol. Reverse transcriptase PCR was performed using a M-MLV reverse transcriptase (Promega, USA). Real-time PCR reactions were performed using SYBR^®^ Premix Ex Taq™ (Takara, Japan) and 300 nmol/L of each primer. Amplification was performed according to the manufacturer’s protocol of the 7500 Fast Real-Time PCR Systems (Applied Biosystems, USA). Primer sequences used are listed in Supplemental information.

### Immunoprecipitation and immunoblotting analysis

Immunoprecipitation was performed with the lysates from indicated cultured cells and followed by the immunoblotting with corresponding antibodies. Briefly, after trypsinizing, the cells were harvested and washed twice with cold PBS. Cell pellets were resuspended and lysed in the buffer (50 mM Tris-HCl, pH 7.4, 150 mM NaCl, 1% Triton X-100, 5 mM EDTA, 1 mM NaVO3, 50 mM NaF and protease inhibitor cocktail). Then the lysates were centrifuged at 13,000*g* to remove the cell debris. Supernatant was transferred a prechilled microcentrifuge tube. Protein concentration was determined using the BCA Protein Assay Kit (Pierce) according to the instruction of manufacturer. 1 mg of protein were incubated with indicated antibodies overnight and then mixed with protein A or protein G-agarose beads (Santa Cruz, USA). Immunocomplexes were collected by centrifugation at 1000*g* and resolved on SDS-PAGE gel and subsequently transferred to polyvinylidene fluoride membranes (Millipore). The blots were blocked with 5% nonfat milk followed by the incubation of primary antibodies and HRP-conjugated secondary antibodies. Blots were developed using SuperSignal West Pico (Thermo Scientific, USA) and detected by Tanon 6600 Luminescent Imaging Workstation.

### Luciferase reporter gene assay

The transcriptional activation of p53 in SK-Hep1 cells was measured using Dual-luciferase Assay Kit (Promega) on GloMax 20/20 luminometer (Promega, E1910) following the manufacturer’s instruction. In detail, the cells were plated, in triplicates, at a density of 20,000 cells/well in a volume of 500 μL in 24-well plates. After transfected with indicated plasmids for 48 h, the cells were washed by cold PBS and lysised by passive lysis buffer. The relative levels of luciferase activity were normalized to the levels of untreated cells and to the levels of luciferase activity of the Renilla control plasmid in each group.

### Flow cytometry analyses for cell death

Cell death was detected with an annexin V-fluorescein isothiocyanate (FITC)/PI kit following the manufacturer’s instruction. After treatment, cells were trypsinized, collected by centrifugation, washed with PBS, and resuspended at a density of 5 × 10^5^ cell/mL with 1× annexin V binding buffer. Then 5 µL annexin V-FITC conjugates and 5 µL PI solution were added and incubated for 15 min in the dark. Finally, cells were incubated with 1× annexin V binding buffer and analyzed within 1 h by flow cytometric analysis (BD FACS Aria SORP, USA). At least 30,000 cells were analyzed to determine the percentage of apoptotic cells.

### LentiCRISPR/cas9 screening and sequencing

LentiCRISPR v2 plasmids pool was a gift from Feng Zhang lab (Addgene plasmid # 52961) and the screening strategy was performed as previously described [20]. In detail, lentiCRISPR v2 plasmids pool was packaged into lentivirus by transfecting HEK 293T cells. Then 1 × 10^8^ SK-Hep1 cells were infected with the lenti-CRISPR library at 0.2 multiplicity of infection. Two days later, puromycin (5 μg/mL) was added to the medium and continuously cultured for another 2 days. After trypsinizing, the survived cells were harvested, then seeded in 10 cm dishes with 5 × 10^6^ cells per dish. Twelve hours later, cells were treated with Aza (1 μM) for 2 days, then the survived cells were harvested to extract genomic DNA for whole genome sequencing by Illumina sequencer with HiSeq instrument. Sequencing data analysis and management were performed with BaseSpace Sequence Hub.

### Immunoprecipitation and analysis by liquid chromatography-mass/mass

Immunoprecipitation was performed with the lysates from indicated cultured cells and followed by liquid chromatography-mass/mass. The process of immunoprecipitation had been described in additional experimental procedures. These total fractions from SDS-PAGE gel were processed as described [40], such as reductive alkylation, trypsin digestion and peptide extraction. The peptides were analyzed by liquid chromatography mass spectrometry/mass spectrometry on a Q Exactive mass spectrometer (Thermo Fisher Scientific, Waltham, MA).

### CRISPR-cas9 mediated *LOXL4* gene editing through homology-directed repair

The pSpCas9(BB)-2A-GFP (pX458) vector (Addgene, #48138) expressing Cas9 and containing a cloning site for the sgRNA sequence was digested with BbsI (NEB). The sgRNA-1, -2, and -3 were synthesized, annealed, and ligated to the pX458 plasmid. The sequences of designed sgRNAs were:

5′-CACCTAGGCTGCTGGGACACCTAC-3′ (sgRNA-1, sense);

5′-AAACGTAGGTGTCCCAGCAGCCTA-3′ (sgRNA-1, anti-sense);

5′-CACCGGCATGACATTGATTGCCAG-3′ (sgRNA-2, sense);

5′-AAACCTGGCAATCAATGTCATGCC-3′ (sgRNA-2, anti-sense);

5′-CACCTGACATTGATTGCCAGTGGG-3′ (sgRNA-3, sense);

5′-AAACCCCACTGGCAATCAATGTCA-3′ (sgRNA-3, anti-sense);

The correct insertion of the sgRNA sequences was confirmed using Sanger sequencing.

Double-stranded homology-directed repair (HDR) template was prepared by PCR amplification. 5′HA was amplified with specific primers (primer-HA1 5′-CCTCTGAATTCCCCACGCATTGT-3′ and primer-HA2 5′- CACCCACTGGCATCGAATACGATGCCGTAGGTGTCCCAGCAG-3′). 3′HA was amplified with specific primers (primer-HA3 5′-CACCTACGGCATCGTATTCGATGCCAGTGGGTGGATATCACAG-3′ and primer-HA4 5′-TGTGTGGCAGTTGTGCAGCCAG-3′). Then 5′HA and 3′HA fragments were used to assemble HDR template with primer-HA1 and primer-HA4 by PCR. The PCR product was extracted with phenol:chloroform:isoamylalcohol and then with chloroform, before isopropanol precipitation 2 h at −20 °C. The DNA pellet was washed with 70% ethanol three times, dried by vacuum and dissolved in water. DNA concentration was determined by Nanodrop (Thermo Fisher Scientific, USA).

Cells were co-transfected with pX458 and HDR template. Twenty-four hours later, green flourescent protein (GFP)-positive cells were sorted into single cell by fluorescence-activated cell sorting. After 14 days culture, cells were trypsinized and genomic DNA were extracted. The target site (sequence around *LOXL4* mutation site) was amplified by PCR with specific primers (5′-TCCTTACAGGACTGCAGCG-3′ and 5′-TCCCTAACAGGACAGCTCCT-3′). Then the PCR product was sequenced to verify the mutation.

### In vivo tumor xenograft

Five- to seven-week-old BALB/c nude mice were purchased from the Shanghai Institute of Material Medica, Chinese Academy of Sciences (Shanghai, China) and grouped into five mice/group. Indicated SK-Hep1 cells (2 × 10^6^) were subcutaneously injected into the left dorsal part of mice. The tumors were measured every three days with microcalipers and tumor volume was measured using tumor length (L) and width (W) and calculating the volume with the formula LW^2^/2. Tumors were dissected and snap-frozen for molecular biology analyses.

### Clinical samples and IHC staining

This study was approved by the institutional review board of Ruijin Hospital, Shanghai Jiaotong University. Each patient signed the informed consent. The diagnoses of these liver cancer samples were verified by pathologists. The use of these tissue materials for research was approved by the Ruijin Hospital. We compared survival durations of 83 patients, all of whom received curative resection, with low (0–2 staining score) versus high (3–5 staining score) of LOXL4 and p-p53(S15). The staining scores were on a scale of 0–5, corresponding to the staining intensity of tumor tissue. Every section was randomly captured for 6 times at different regions and the average of the staining scores form six regions represents the sample.

### Statistical analysis

All results were presented as the mean ± standard error of mean, unless stated otherwise. The two-tailed unpaired student’s *t* test was performed to compare the differences between treated groups relative to their paired controls. One-way ANOVA was used to analyze tumor growth data. *p* < 0.05 was considered significant; *p < 0.05; **p < 0.01; ***p < 0.001. Pearson’s correlation analyses were used to calculate the regression and correlation between two groups. DAVID Functional Annotation Bioinformatics Microarray Analysis was used to perform Enrichment of Gene Ontology terms among LOXL4 interactors identified in SK-Hep1. Statistical analyses were performed using GraphPad Prism 7 (GraphPad Software, Inc.)

## Supplementary information

all supplementary data
