# Supplementary material for: Derepression of LOXL4 inhibits liver cancer growth by reactivating compromised p53
Source: Cell Death Differ. 2019 Feb 6;26(11):2237–52. doi: 10.1038/s41418-019-0293-x (PMC6889417; doi:10.1038/s41418-019-0293-x)
Supplement: Supplementary file 1 — all supplementary data [file 41418_2019_293_MOESM1_ESM.docx]

**Supplementary Material**

**Supplemental Figure and Legend**

**
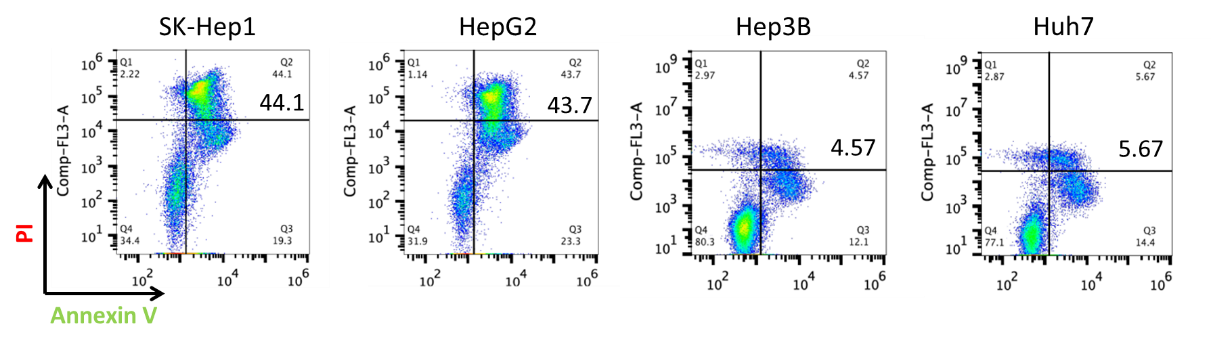
**

**Figure S1. 5-aza-CR induced apoptosis in SK-Hep1 and HepG2 cells but not in Hep3B and Huh7 cells.** Cells were treated with 5-aza-CR (5 μM) for 16 h before collection and then a FACS analysis was performed according to the operation manual of the PI and anti-Annexin V double staining kit. Experiments were independently performed three times.

**
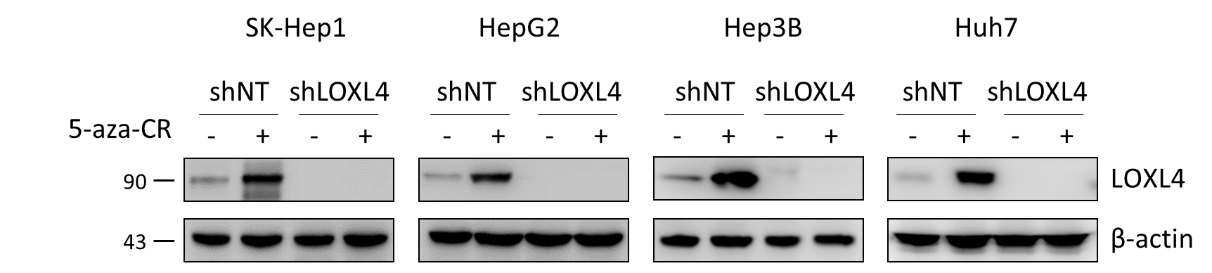
**

**Figure S2. Validation of LOXL4 knockdown efficiency with or without 5-aza-CR treatment by western blotting.** The indicated cells were treated with or without 5 uM 5-aza-CR for 16 h, then the cells were lysted and protein were extracted for western blotting analysis.

**
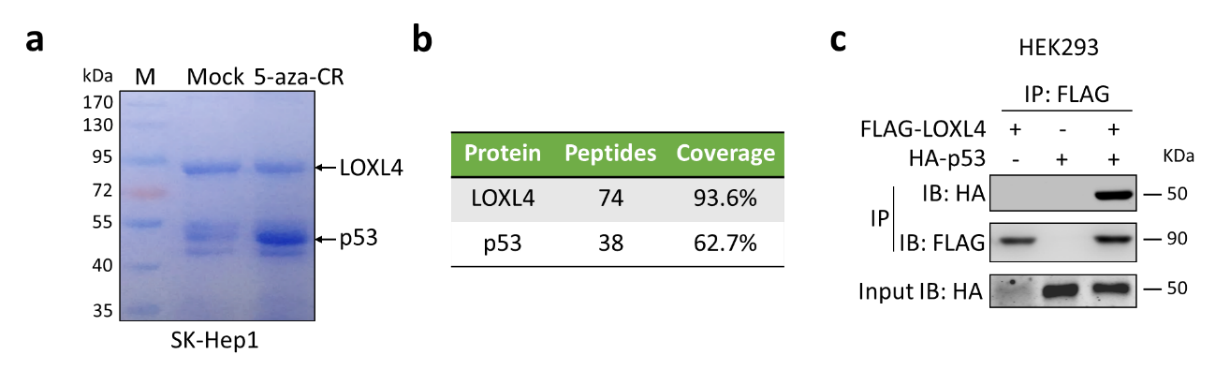
**

**Figure S3. LOXL4 interacts with wild-type p53.** **a, b** LC-MS/MS identified p53 is a probable candidate of LOXL4. p53 band specially appears after 5-aza-CR treatment (5 μM, 8 h). **c** co-immunoprecipitation of LOXL4 and p53 in HEK293 cells after co-transfected with FLAG-LOXL4 and HA-p53 plasmids. M means protein markers.


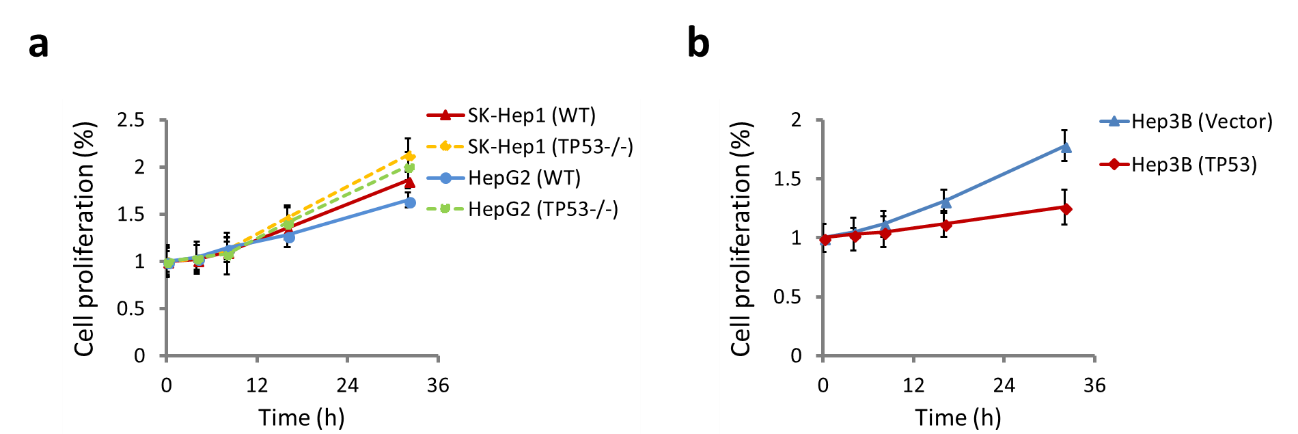


**Figure S4. Cell proliferation of SK-Hep1, HepG2 and Hep3B cells.** **a, b** indicated cells were seeded and cultured for indicated time. Then the cell number was measured with a standard CCK-8 assay. The 0 h value was set to 1.0; data were from three independent experiments performed in triplicate; error bars represent SEM.

**
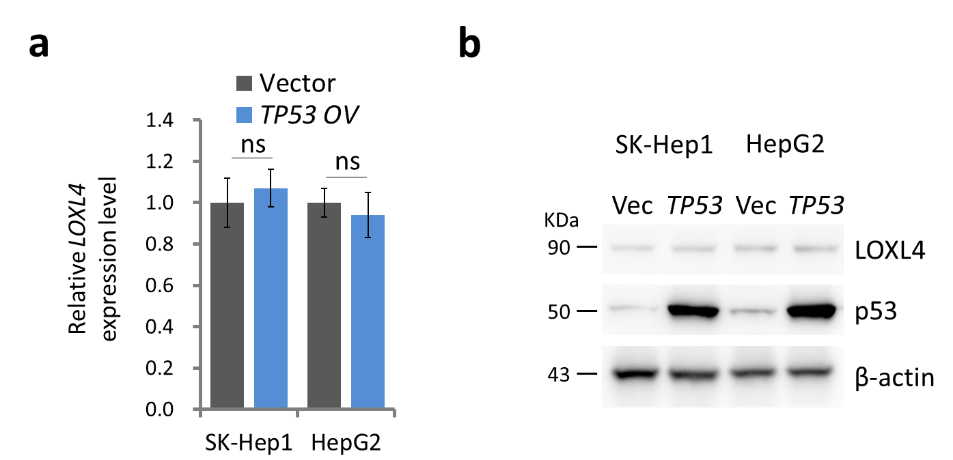
**

**Figure S5. Overexpressing of *TP53* does not increase the mRNA level and protein level of LOXL4.** SK-Hep1 and HepG2 cells were overexpressed WT TP53 for 48 h, then the mRNA was extracted for q-RT-PCR analysis and the cell lysates were used for western blot analysis.

**
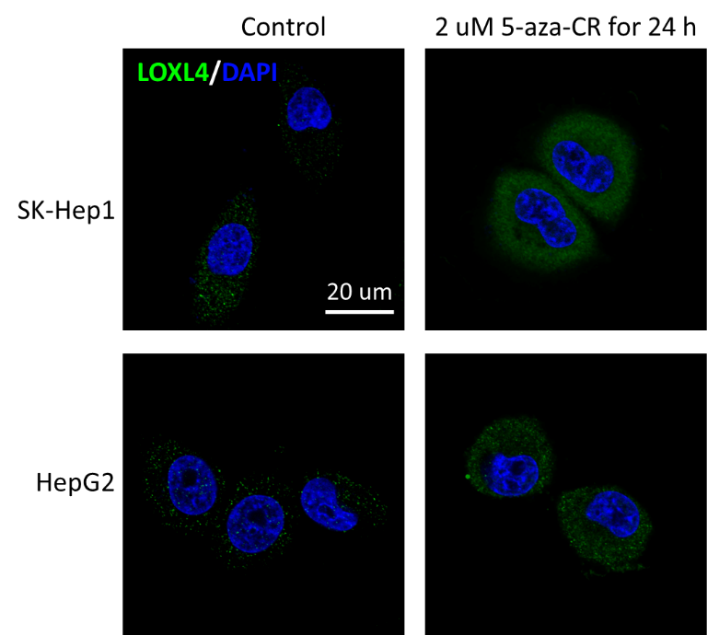
**

**Figure S6. Immunofluorescence staining of LOXL4.** SK-Hep1 and HepG2 cells were untreated or treated with 2 uM 5-aza-CR for 24 h. Immunofluorescence was performed with anti-LOXL4 antibody (1:100 dilution).

**
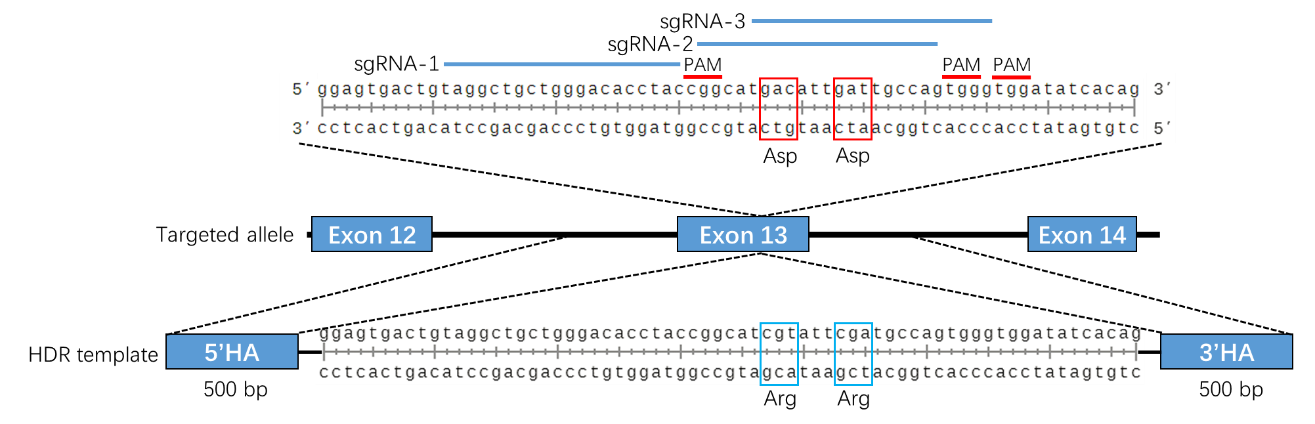
**

**Figure S7. Schematic of the Cas9-sgRNA-targeting sites in *LOXL4* gene.** Blue lines label the sgRNA-targeting sequences and red lines represent PAM sequences. HA means homology arms (500 bp).

**Primers used in this study**

1. Primers used for qRT-PCR

GAPDH –F: GAGTCAACGGATTTGGTCGT

GAPDH –R: TTGATTTTGGAGGGATCTCG

PIG3-F: 5’- CCATGCAGGACTGAGTGGTG-3’

PIG3-R: 5’- CTGCTCCAAGCTTTTCTGCC-3’

PUMA-F: 5’-GCCAGATTTGTGAGACAAGAGG-3’

PUMA-R: 5’-CAGGCACCTAATTGGGCTC-3’

BAX-F: 5’-CCCGAGAGGTCTTTTTCCGAG-3’

BAX-F: 5’-CCAGCCCATGATGGTTCTGAT-3’

GPX1-F: 5’- CAGTCGGTGTATGCCTTCTCG-5’

GPX1-R: 5’- GAGGGACGCCACATTCTCG -5’

SOD2-F: 5’-GGAAGCCATCAAACGTGACTT-3’

SOD2-R: 5’-CCCGTTCCTTATTGAAACCAAGC-3’

TIGAR-F: 5’ -CCATGTGCAATCCAGAGATG-3’

TIGAR-R: 5’ -CCTTACCAGCCACTCTGAGC-3’

Sestrin 1-F: 5’- TGCTTTGGGCCGTTTGGATAA-3’

Sestrin 1-R: 5’- TGTAGTGACGATAATGTAGGGGT -3’

2. LOXL4 shRNA:

609: 5’-GGAAAGTCTGGGATCTGAAGA-3’

1173: 5’-GGTGCAATGTCCCTAACATGG-3’

3. Cloning Homo sapiens LOXL4:

LOXL4-F: 5’-ATGGAATTCGCTAGCGGATCC atggcgtggtccccaccagcc-3’

LOXL4-R: 5’-ATGTCGACCTCGAGTGCGGCCGCtta gatgaggttgttcctga-3’
